# Comparison of Herbicide Responses Between Root Re-Growth in *Lemna aequinoctialis* and Root Development During Turion Germination in *Spirodela polyrhiza*

**DOI:** 10.3390/toxics14090791

**Published:** 2026-09-07

**Authors:** Hojun Lee, Taejun Han, Jihae Park

**Affiliations:** 1 Marine@UGent Korea, 119-5, Songdomunhwa-ro, Incheon 21985, Republic of Korea; hojun.lee@ugent.be (H.L.); taejun.han@ghent.ac.kr (T.H.); 2 Bio Environmental Science and Technology (BEST) Lab, Ghent University Global Campus, 119-5, Songdomunhwa-ro, Incheon 21985, Republic of Korea; 3 Department of Animal Sciences and Aquatic Ecology, Ghent University, Coupure Links 653-Block F, B-9000 Gent, Belgium; 4 Center for Green Chemistry and Environmental Biotechnology, Ghent University Global Campus, 119-5, Songdomunhwa-ro, Incheon 21985, Republic of Korea

**Keywords:** duckweed, root length, root re-growth, turion germination, *Lemna aequinoctialis*, *Spirodela polyrhiza*, herbicides, bioassay comparison, test standardization

## Abstract

Root length is increasingly used as a rapid endpoint in duckweed ecotoxicology, yet biologically distinct assays are often treated as equivalent. Two 72 h root-length tests are now in use: root re-growth after excision in mature *Lemna aequinoctialis* fronds, and root development during germination of rootless *Spirodela polyrhiza* turions exposed directly to the test solution. Both begin from zero initial root length, but for different biological reasons. We asked whether they produce comparable herbicide responses. Thirteen herbicides were drawn from a screening campaign in which both assays were run under matched exposure conditions. Nine compounds returned quantified EC_50_ estimates (with the *L. aequinoctialis* coefficient of variation below 40%) and formed the primary comparison. EC_50_ ratios (*S. polyrhiza*/*L. aequinoctialis*) ranged from 0.75 to 430 (median 2.76). Five compounds agreed within about three-fold, whereas four compounds spanning several target-site classes diverged by 74- to 430-fold, and potency rankings were not significantly correlated (Spearman ρ = 0.43, *p* = 0.24). For the common reference toxicant, the two assays differed by only about two-fold, and in the opposite direction; comparable reference-toxicant performance therefore did not predict comparable herbicide responses. The two assays should not be treated as interchangeable root-length tests. However, they differ not only in the biological origin of the measured root but also in species and developmental starting condition, and the present design cannot separate these factors; the observed differences therefore demonstrate assay-level non-equivalence without identifying its biological cause.

## 1. Introduction

Herbicides are designed to disrupt processes that are essential to plants, and they may therefore affect non-target aquatic macrophytes when they reach surface waters through run-off, drainage and accidental discharge [1,2]. Duckweeds (Lemnaceae) have been used for decades in aquatic ecotoxicology because of their small size, rapid vegetative growth, morphological homogeneity, ease of handling and suitability for tests conducted in small volumes of water [3,4,5]. They are also the aquatic macrophytes on which prospective herbicide risk assessment has principally relied [6,7].

Conventional duckweed toxicity tests evaluate frond number, frond area, biomass or relative growth rate over exposure periods of seven days or longer, as specified in ISO 20079 and OECD Test Guideline 221 [8,9]. Alongside these growth endpoints, root length has emerged as a rapid and sensitive indicator of chemical stress [10,11,12].

Duckweed roots are in continuous contact with dissolved contaminants, and root elongation, being the combined outcome of cell division and cell expansion, can respond to adverse conditions over short periods. Root length is straightforward to quantify by image analysis and has been reported to be at least as sensitive and more precise than frond-based endpoints for metals, mining effluents and herbicides [10,11]. It can therefore support a concentration–response relationship within 72 h, well before conventional whole-plant growth tests reach their measurement point.

Because pre-existing roots differ in length between individuals, the initial condition of a root-length test is itself a source of variance. A root re-growth test was developed in which all roots of mature *Lemna* fronds are excised immediately before exposure, so that the initial root length is standardized to zero and the length of newly produced roots is measured after exposure [11]. The approach requires a short exposure period and a small test volume, permits non-axenic plant material, and has since been examined in an interlaboratory comparison involving ten institutes and formalized as an international test method for *Lemna minor* [13,14]. In this context, root re-growth refers operationally to the reappearance and elongation of roots after complete excision from mature vegetative fronds; it should be distinguished from the continued elongation of an intact root, and no measurement of meristem activity, hormone signalling or wound response is implied.

An initial root length of zero can also arise without excision. *Spirodela polyrhiza* forms dormant turions, starch-rich vegetative propagules produced in response to declining temperature and photoperiod, which sink and remain quiescent until conditions become favourable [15,16,17]. Turions carry no roots, can be stored for months at low temperature and can be used directly as test material, which removes the need to maintain a stock culture; these properties underlie the standardized *Spirodela* microbiotest [18,19] and have been exploited in root-length assays with metals and with paraquat [20,21,22]. When rootless turions are placed directly into the test solution, germination and the formation of the first roots both take place in the presence of the test substance. The initial root length is then zero without any excision, and the measured response is root emergence and elongation de novo during germination rather than re-growth after excision.

**Figure 1:**
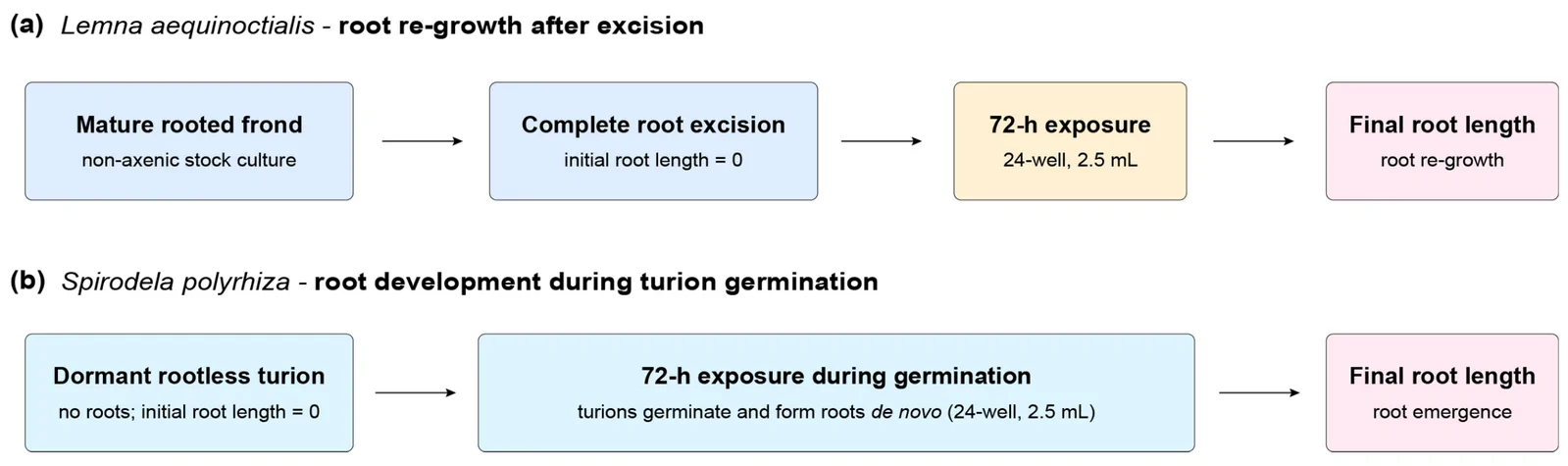
Design of the two 72 h root-length assays. (**a**) In the *L. aequinoctialis* assay, the initial root length of zero is created by excising the roots of a mature frond immediately before exposure, and the measured response is root re-growth. (**b**) In the *S. polyrhiza* assay, the initial root length of zero is inherent to the rootless turion; turions are placed directly into the test solution and germinate during the exposure, so the measured response is root emergence and elongation de novo. Exposure period, test vessel, test volume, irradiance and measured endpoint are the same in the two assays.

The two assays are operationally similar to an unusual degree. Both begin from a root length of zero, both quantify root elongation over 72 h in 24-well plates using a few millilitres of test solution, both are read by image analysis, and both express toxicity as an EC_50_ for root-length inhibition (Figure 1). What differs is the biological origin of the measured root and the species. Whether the two designs produce comparable herbicide responses has not been established. Earlier comparisons between *Spirodela* and *Lemna* tests used frond-based endpoints and reported broadly similar sensitivities across mixed chemical classes [18], while comparisons of turions with active fronds of the same species found turions to be the more tolerant material for several metals [20]. Neither addresses the question raised here, in which the endpoint and the exposure format are held constant and the starting condition is not.

This question is not confined to the two methods examined here. Root length now appears as a measurement endpoint in standardized and candidate duckweed test methods that differ in the test species and in the developmental state of the test organism [13,14,19], and the case for extending such a method to a new species or a new starting condition is normally made on the grounds that the endpoint, the exposure duration and the test format are unchanged. If two assays that appear operationally equivalent respond differently to herbicides, similarity in these design features may not guarantee comparable ecological interpretation of the resulting effect concentrations. Standardizing a measurement endpoint is thus not the same as standardizing a complete test system, and the distinction is central to the comparison made here.

This study compared 72 h EC_50_ values for inhibition of root length in mature *L. aequinoctialis* fronds after root excision and in *S. polyrhiza* turions germinating in the test solution. Herbicides were retrieved from a broader screening dataset in which both assays had been applied to the same compounds under matched exposure conditions. We tested the hypothesis that two duckweed assays sharing the same root-length endpoint and exposure design nevertheless produce non-equivalent herbicide responses. Three specific questions were addressed: whether the two assays agree in the magnitude of the EC_50_; whether they rank herbicide toxicity in the same order; and whether the relative sensitivity observed for the reference toxicant of the two test methods carries over to herbicides.

## 2. Materials and Methods

### 2.1. Test Organisms and Culture

*Lemna aequinoctialis* Welw. (formerly *L. paucicostata* Xu et al. [23]) was maintained non-axenically in Steinberg medium [24] in glass vessels at 25 ± 1 °C under continuous cool-white light of 30–40 µmol photons m^−2^ s^−1^. The medium was renewed weekly and adjusted to pH 6.9 ± 0.1. Healthy colonies of two to three fronds, uniformly dark green and free of chlorosis or necrosis, were selected as test material.

*Spirodela polyrhiza* (L.) Schleiden was cultured under the same conditions, and turion formation was induced by transferring cultures to reduced temperature. Detached turions were collected from the bottom of the culture vessels and stored in Steinberg medium at 0–5 °C in darkness for two to six months. Before testing, intact turions of uniform size, without visible roots or fronds, were selected and transferred directly to the test solutions; no pre-germination step preceded exposure.

### 2.2. Test Chemicals

Thirteen herbicides were available in the screening panel (Table 1). All chemicals were of analytical grade and obtained from Sigma-Aldrich (St. Louis, MO, USA). The panel spanned five herbicidal modes of action (HRAC 2020 classification): six photosystem II inhibitors acting at the D1 Ser264 site (HRAC group 5; atrazine, diuron, isoproturon, linuron, simazine and terbutryn), four inhibitors of very-long-chain fatty acid elongases (HRAC group 15; alachlor, butachlor, metolachlor and thiobencarb), the photosystem II inhibitor bentazone acting at a distinct site (HRAC group 6), the synthetic auxin MCPA (HRAC group 4) and the acetolactate synthase inhibitor penoxsulam (HRAC group 2). Stock solutions were prepared in dimethyl sulfoxide (DMSO) and used to prepare the highest test concentration for each herbicide. The remaining concentrations were prepared by sequential two-fold dilution with test medium. Consequently, the DMSO concentration decreased proportionally across the dilution series, with a maximum final concentration below 0.1% (*v*/*v*) at the highest herbicide concentration. The negative control consisted of test medium without herbicide or DMSO; a separate DMSO solvent control was not included. The pH of the test medium was 6.9 ± 0.1. The compound- and assay-specific definitive concentration series are provided in Table S1. Range-finding tests preceded the definitive tests. In the definitive tests, a two-fold dilution series of five concentrations plus a negative control was used, and the pH of the highest concentration was adjusted to 6.8–7.0. All concentrations are nominal; exposure concentrations were not verified analytically. The highest nominal test concentrations for some herbicides approached or exceeded their reported aqueous solubilities (Table S1). Potential exposure-related uncertainties associated with precipitation, sorption, and degradation are discussed in Section 4.5.

### 2.3. Root Re-Growth Assay with L. aequinoctialis

Immediately before exposure, all roots were excised from the selected fronds with stainless-steel scissors, setting the initial root length to zero [11,14]. One rootless plant was transferred to each well of a 24-well plate (well diameter 15.6 mm) containing 2.5 mL of test solution, with four wells per concentration and three replicate plates, giving twelve individuals per concentration. Plates were covered, sealed at the sides with parafilm and incubated at 25 ± 1 °C under continuous white light of 100 ± 10 µmol photons m^−2^ s^−1^ for 72 h without renewal of the test solution. Plants were positioned so that they did not adhere to the well wall or lid.

### 2.4. Turion-Germination Root Assay with S. polyrhiza

Rootless turions were transferred directly from cold storage to 24-well plates containing 2.5 mL of test solution per well, three turions per well and four wells per concentration, giving twelve individuals per concentration. No root excision was performed. During the 72 h exposure the turions germinated and formed roots de novo, so the roots measured at the end of the test were those that emerged and elongated during germination in the presence of the test substance. Incubation conditions, sealing and exposure duration were identical to those used for the *L. aequinoctialis* assay.

Exposure conditions were therefore matched between the two assays, but organism loading was not: one *Lemna* frond and three *Spirodela* turions were placed in each well, in accordance with the respective assay protocols, and the two test materials differ in size and biomass. This difference is inherent to the two methods as they are performed and is considered further in Section 4.5.

### 2.5. Measurement of Root Length

After 72 h, the plants were lifted with tweezers and placed on glass slides with the frond in contact with the glass and the root-bearing surface uppermost. Because the material is wet, the roots could be straightened with light manipulation. Slides were photographed over a light plate together with a scale bar. Root length was measured in ImageJ (version 1.54p, National Institutes of Health, Bethesda, MD, USA) after calibration against the scale bar in every image; the longest root of each individual was measured, as is conventional in duckweed root tests [10,11,21].

### 2.6. Data Treatment and Statistics

Root lengths were expressed as percentage inhibition relative to the negative control. For the *S. polyrhiza* assay, the well was treated as the experimental unit, as the three turions within a well shared the same exposure environment and were not independent. Root lengths of the three turions were averaged within each well to give four independent well-level observations per concentration, and the mean response at each concentration was calculated from these four well-level means. A single EC_50_ estimate for each herbicide was then obtained by linear interpolation (ToxCalc 5.0, Tidepool Scientific, McKinleyville, CA, USA). Because this procedure yields a single EC_50_ estimate rather than independent replicate estimates, an EC_50_-level coefficient of variation and confidence interval were not calculated for the *S. polyrhiza* assay. For the *L. aequinoctialis* assay, EC_50_ values were estimated independently for each of the three replicate determinations by linear interpolation using the same software. The value reported in Table 1 is the mean of the three replicate estimates; the coefficient of variation (CV) was calculated as the standard deviation of the three estimates divided by their mean and multiplied by 100, and the 95% confidence interval was calculated using Student’s t distribution as the mean ± t_0.975,2_·SD/√3. Linear interpolation was used consistently for EC_50_ estimation in both assays.

Historical method-performance ranges were available for control root length and for periodically determined copper sulfate EC_50_ values. The historical performance values (mean ± SD) are 18.910 ± 10.238 mm for control root length and 0.306 ± 0.103 mg L^−1^ for the copper sulfate EC_50_ in the *L. aequinoctialis* assay, and 15.220 ± 5.887 mm and 0.131 ± 0.032 mg L^−1^ in the *S. polyrhiza* assay. These are laboratory performance data compiled for each of the two methods during their development; they are not values from the ten-institute interlaboratory comparison of the *L. minor* root re-growth test [13], which used a different test species. The control root lengths obtained in the definitive herbicide experiments are reported separately in Table S2.

Compounds were assigned to the primary comparison set when quantified, non-censored EC_50_ values were obtained in both assays and the CV among the independently replicated *L. aequinoctialis* EC_50_ estimates was below 40%. The 40% CV threshold was used as an operational precision criterion for defining the primary comparison set and was not treated as a formal test-validity criterion [25]. Compounds with quantified EC_50_ values in both assays but exceeding this precision criterion in the *L. aequinoctialis* assay were retained in the supporting set. Compounds for which either assay returned only a censored EC_50_ (greater than the highest tested concentration) were excluded from ratio-based quantitative comparisons and are listed in Table 1 for completeness. As a sensitivity check, the classification was also examined using a 30% CV threshold for the independently replicated *L. aequinoctialis* estimates (Section 3.5).

Agreement between assays was expressed as the ratio *R* = EC_50,*Spirodela*_/EC_50,*Lemna*_, so that *R* > 1 indicates that the *Lemna* assay is more sensitive. Concordance of potency ranking was assessed by Spearman rank correlation. Analyses were performed in Python 3.12 with SciPy.

## 3. Results

### 3.1. Composition of the Comparison Sets

Of the thirteen herbicides in the panel, nine returned quantified EC_50_ values in both assays and had a CV below 40% among the independently replicated *L. aequinoctialis* EC_50_ estimates, forming the primary comparison set: alachlor, bentazone, butachlor, linuron, MCPA, metolachlor, terbutryn, penoxsulam and isoproturon (Table 1). Diuron also returned quantified EC_50_ values in both assays but was retained in the supporting set because the CV among the three replicate *L. aequinoctialis* EC_50_ estimates exceeded 40%. For atrazine, simazine and thiobencarb, the *S. polyrhiza* EC_50_ remained above the highest concentration tested, and these compounds were therefore not included in ratio-based quantitative comparisons.

### 3.2. Magnitude of the Difference Between Assays

**Figure 2:**
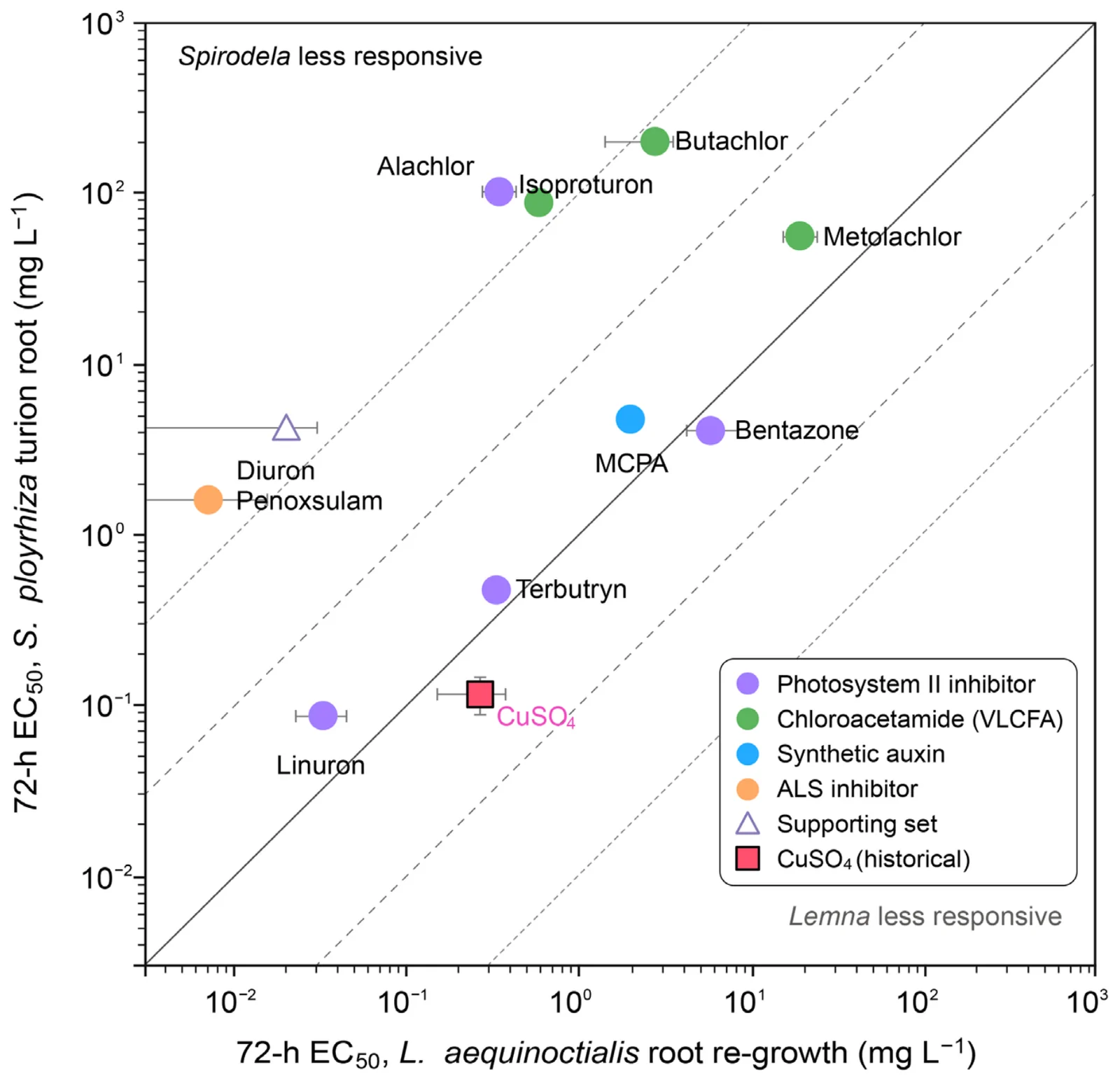
Seventy-two-hour EC_50_ values for root-length inhibition in the two assays. Filled symbols indicate primary-set compounds, and the open triangle indicates the supporting-set compound (diuron); points are coloured by herbicide target site. Horizontal error bars represent 95% confidence intervals for the *L. aequinoctialis* EC_50_ estimates. For diuron, the lower confidence bound was negative and is therefore truncated at the lower plotting limit of the logarithmic axis. The red square indicates historical copper sulfate performance values, with error bars representing historical standard deviations. The solid line indicates the 1:1 relationship; dashed and dotted lines indicate 10-fold and 100-fold deviations, respectively.

**Figure 3:**
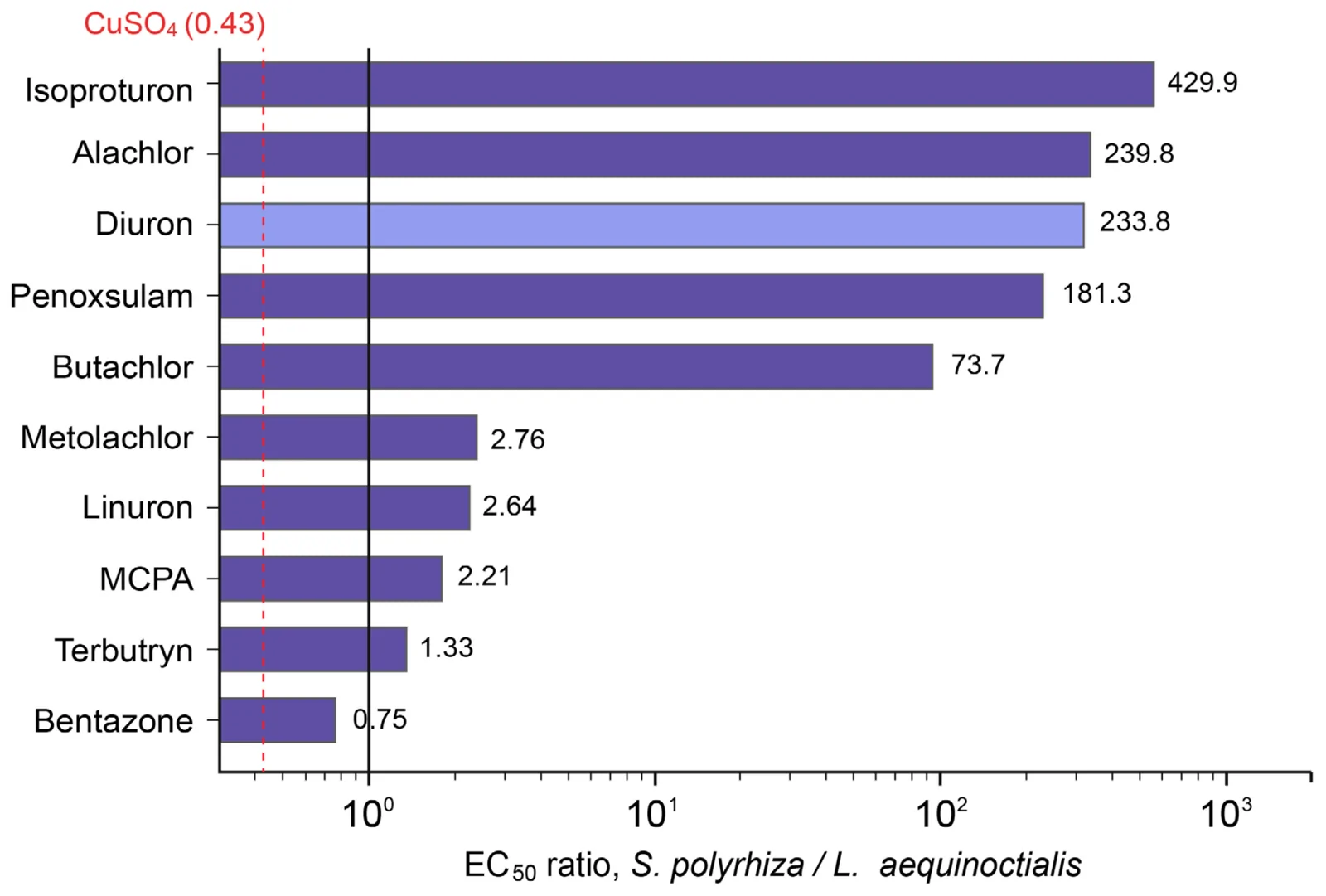
Ratio of the two EC_50_ values (*R* = EC_50,*Spirodela*_/EC_50,*Lemna*_) for the ten compounds with quantified EC_50_ values in both assays. Values above unity indicate that the *L. aequinoctialis* root re-growth assay was more sensitive. Primary-set compounds are shown as solid bars and the supporting-set compound (diuron) as a pale bar. The dashed red line marks the corresponding ratio (R = 0.43) for the historical copper sulfate performance values.

Within the primary set, the two assays did not differ by a single offset (Figure 2). The ratio *R* ranged from 0.75 to 430, a spread of nearly three orders of magnitude, with a median of 2.76 and a geometric mean of 14.1. Bentazone was the only compound for which the *S. polyrhiza* assay was more sensitive (*R* = 0.75). Terbutryn (1.33), MCPA (2.21), linuron (2.64) and metolachlor (2.76) differed by less than three-fold. In contrast, butachlor (73.7-fold), penoxsulam (181-fold), alachlor (240-fold) and isoproturon (430-fold) showed substantially larger differences, with the *L. aequinoctialis* root re-growth assay being more sensitive in every case. Diuron, retained in the supporting set, differed by 234-fold (Figure 3).

Figure 2 shows the same result geometrically. The five closely agreeing compounds cluster on the 1:1 line, whereas the four strongly divergent compounds depart from it progressively: butachlor lies near the 100-fold band and penoxsulam, alachlor and isoproturon lie beyond it, with diuron from the supporting set also far from the line. Because diuron exceeded the precision criterion in the *L. aequinoctialis* assay, its large ratio carries greater uncertainty and is not given the same evidential weight as the primary-set compounds. The departure is one-sided. No compound lay more than 1.34-fold below the 1:1 relationship, so that wherever the two assays disagreed appreciably it was always the *L. aequinoctialis* assay that returned the lower EC_50_. Colouring the points by target site shows that the upper-left region of the plot is occupied exclusively by the chloroacetamides, by penoxsulam and by the photosystem II inhibitor isoproturon, whereas the remaining photosystem II inhibitors are distributed on both sides of the 1:1 line.

The largest between-assay differences were not associated with imprecise *L. aequinoctialis* EC_50_ estimates. Alachlor (240-fold) and isoproturon (430-fold), two of the most divergent primary-set compounds, had *L. aequinoctialis* CVs of 0.83% and 6.21%, respectively, and tightly bounded confidence intervals (Figure 2). Conversely, the compounds that agreed most closely were not systematically the most precisely determined.

### 3.3. Concordance of Potency Ranking

**Figure 4:**
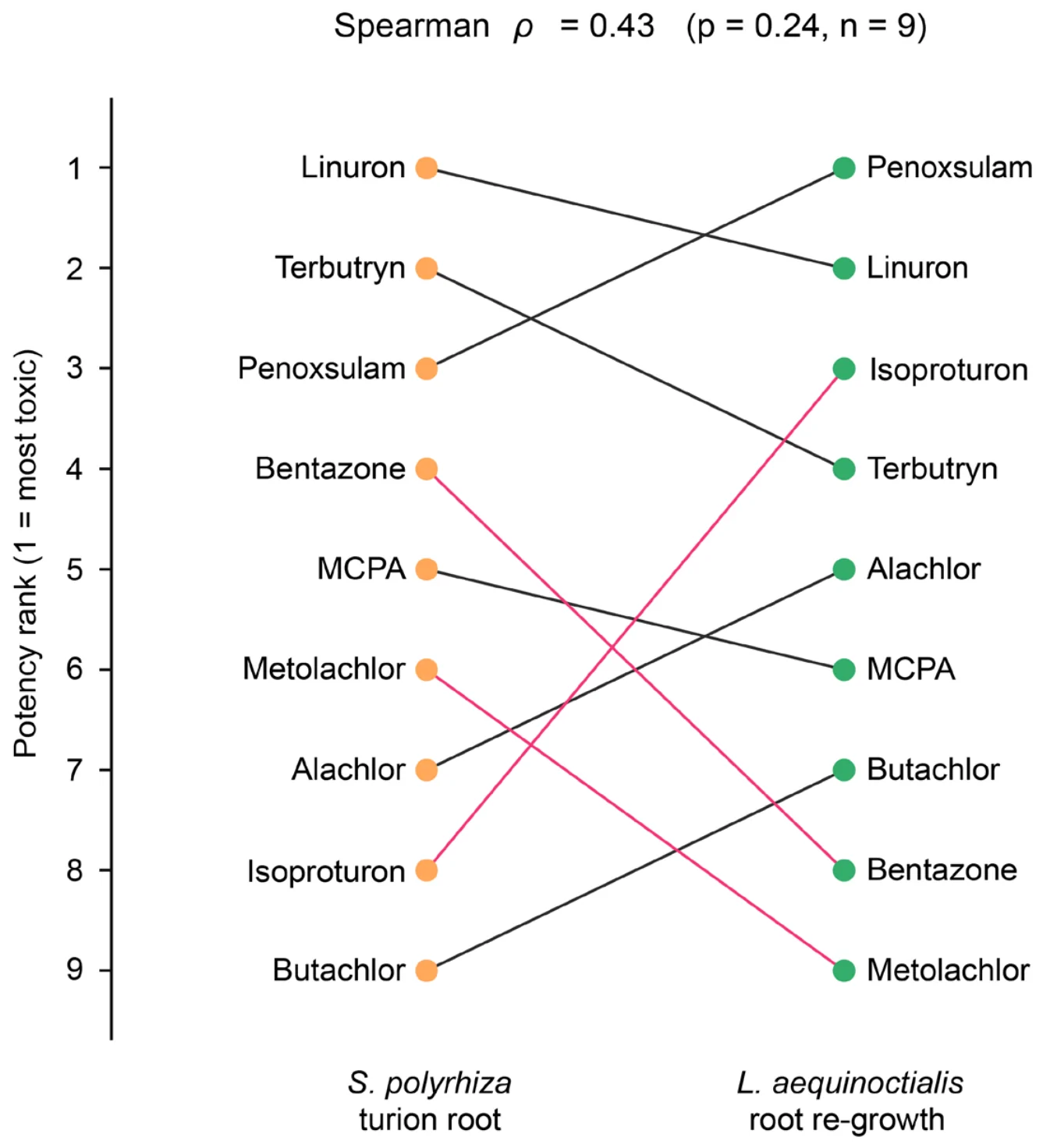
Potency rank of the nine primary-set herbicides in the two assays (rank 1 = most toxic). Lines connect the same compound in the two assays; red lines mark displacements of three or more rank positions. Spearman rank correlation was ρ = 0.43 (*p* = 0.24, *n* = 9).

Ranking the nine primary-set herbicides by potency produced moderate but statistically non-significant concordance between the two assays (Spearman ρ = 0.43, *p* = 0.24, *n* = 9). Of the 36 possible pairwise orderings, 23 were concordant and 13 were discordant between the assays (Table S3). Including diuron, the sole supporting-set compound, gave a similar result (ρ = 0.43, *p* = 0.21, *n* = 10). Given the limited number of compounds, this analysis should be regarded as exploratory. The results therefore indicate that significant concordance of potency ranking was not demonstrated, rather than that the two rankings are independent (Figure 4).

### 3.4. Comparison with the Reference Toxicant

Copper sulfate is the reference toxicant of both test methods. The historical performance values compiled during method development place the *S. polyrhiza* EC_50_ at 0.131 ± 0.032 mg L^−1^ and the *L. aequinoctialis* EC_50_ at 0.306 ± 0.103 mg L^−1^, so that for this compound the *S. polyrhiza* assay is the more sensitive of the two, by a factor of about 2.3 (*R* = 0.43). Only one herbicide in the present dataset lies on that side of unity (bentazone, *R* = 0.75). These reference-toxicant values were compiled from separate control determinations rather than measured alongside the herbicides in a common experiment, so the comparison is indicative of historical method performance rather than a contemporaneous paired result. Within that limitation, the direction of relative sensitivity for copper sulfate is opposite to that observed for almost all of the herbicides examined here (Figure 3).

### 3.5. Sensitivity of the Result to the Precision Criterion

Applying a 30% rather than a 40% CV threshold to the independently replicated *L. aequinoctialis* EC_50_ estimates did not change the composition of the primary comparison set: all primary-set *L. aequinoctialis* estimates had CVs below 30%, whereas diuron remained above either threshold. Thus, the composition of the primary comparison set was insensitive to the choice between these two precision thresholds.

## 4. Discussion

### 4.1. Two Closely Matched Tests Are Not Interchangeable

**Figure 5:**
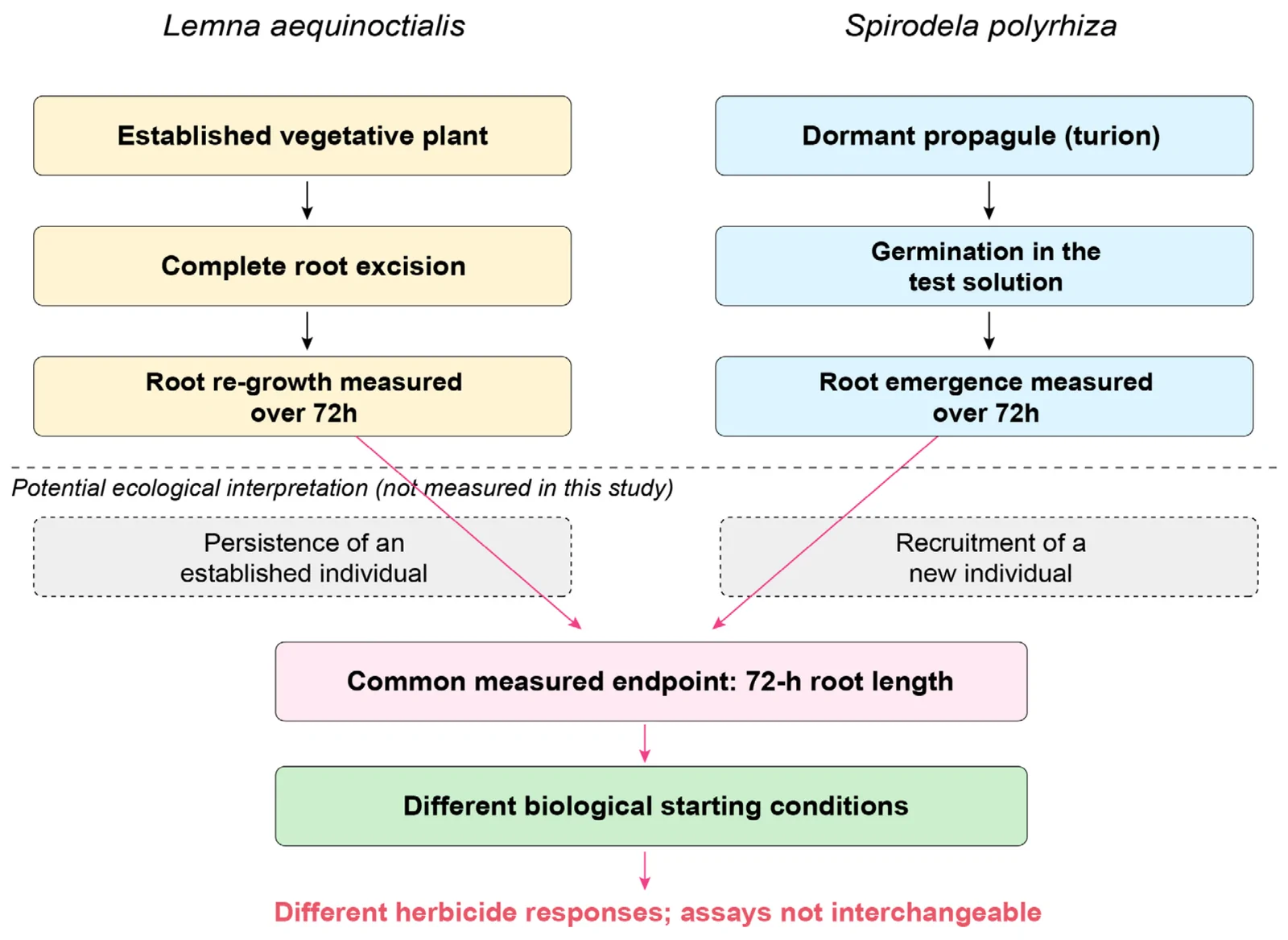
Biological origin of the measured root in the two assays. The solid boxes above the dashed line are the steps actually performed and measured: root re-growth after excision from an established vegetative plant in the *L. aequinoctialis* assay, and root emergence during germination of a dormant propagule in the *S. polyrhiza* assay. The dashed boxes below the line indicate the ecological processes to which the two measurements would correspond; these were not measured in this study and are shown only to indicate a possible interpretation. The measured endpoint is common to both assays, but the starting condition is not.

The two assays compared here are closely matched in several key operational respects. They use the same measured quantity, the same exposure period, the same vessel and volume, the same medium, the same irradiance and temperature, and the same image-based measurement; both were run in one laboratory on one set of chemical stocks; and both begin from an initial root length of zero. Under these conditions, the EC_50_ values still differed by up to approximately 430-fold within the primary comparison set, and significant concordance of potency ranking was not demonstrated. The assays differ, however, in species, developmental starting condition, and the biological origin of the measured root: one measures root re-growth after excision from a mature *L. aequinoctialis* frond, whereas the other measures root emergence during germination from a dormant *S. polyrhiza* turion (Figure 5). These differences are confounded in the present design and therefore cannot be separated as explanations for the observed assay-level differences. The practical conclusion is narrow but firm: an EC_50_ for root-length inhibition is not a property of the endpoint alone, and a value obtained with one of these assays should not be substituted for a value obtained with the other.

This is a stronger statement than the familiar observation that duckweed species differ in sensitivity. Interspecific differences within *Lemna* for herbicides measured on root re-growth are typically within an order of magnitude [1], and comparisons between *Spirodela* microbiotests and *Lemna* growth tests across mixed chemical classes have generally reported similar sensitivity ranges [18]. The differences reported here are larger and, more importantly, they are compound-specific rather than a constant offset between two test systems.

### 4.2. The Difference Is Not a Fixed Offset

If one assay were simply more sensitive than the other, the two could still be used interchangeably after a correction factor, and their rankings would agree. Neither holds. The ratio spans from below unity to more than two orders of magnitude, and the reference toxicant of both methods sits on the opposite side of unity from almost every herbicide. A laboratory comparing the two methods on copper sulfate would find them agreeing within a factor of about two and would have no indication that they can differ by two orders of magnitude on a herbicide. Reference-toxicant testing is a control-chart tool for demonstrating that a test system is behaving as expected; similar or acceptable performance with a common reference toxicant should not be interpreted as evidence that two assays will respond similarly to other substances.

### 4.3. Target-Site Classification Does Not Consistently Correspond to the Divergence

In the present analysis, the compounds that diverged most span several mechanistically distinct target-site classes, including the photosystem II inhibitor isoproturon (430-fold), the very-long-chain fatty acid elongase inhibitor alachlor (240-fold), and the acetolactate synthase inhibitor penoxsulam (181-fold), rather than a single class. Chloroacetamides inhibit very-long-chain fatty acid elongases and act principally on dividing and expanding tissue [26]. It might therefore be tempting to interpret the divergence as evidence that the two assays interrogate different developmental processes, since root re-growth after excision and root emergence during germination place different demands on cell division and elongation. However, the present data do not allow this interpretation to be tested directly. The three chloroacetamides themselves diverged by 2.8-fold, 74-fold and 240-fold, an 87-fold spread within a single target-site class; although two photosystem II inhibitors agreed within a factor of three (terbutryn, 1.33-fold; linuron, 2.64-fold), isoproturon differed by 430-fold and diuron (supporting set) by 234-fold, while bentazone was the only compound for which the *S. polyrhiza* assay was more sensitive. Target site alone therefore does not order the divergence. Any association between the observed pattern and herbicide target site should accordingly be read as hypothesis-generating rather than as mechanistic evidence, since no physiological or biochemical endpoints were measured.

The present data cannot distinguish whether the observed differences arise from species identity, developmental starting condition or other assay-level characteristics. One observation is nonetheless worth recording: in the *S. polyrhiza* assay the test substance is present throughout germination as well as root elongation, yet for most herbicides that assay was the less responsive of the two, so the direction of the difference is not the one that an argument from developmental vulnerability alone would predict. For diuron, the available data do not allow the source of the elevated replicate variability to be identified.

### 4.4. Implications for Standardization and Test Batteries

Root-length tests are moving into formal standardization. The root re-growth principle has been through an interlaboratory comparison and has been adopted as an international method for *L. minor* [13,14], and the turion-based *Spirodela* microbiotest is an established standard for frond growth [19]. Extension of either principle to a further species or a further starting condition is therefore an obvious next step, and it is normally justified on the grounds that the endpoint, the exposure duration and the format are the same. The present results indicate that this justification is insufficient on its own. Validity criteria and reference-toxicant sensitivity ranges are properties of a specific test system, and the substance-specific sensitivity of that system does not transfer with the endpoint.

The corollary is more constructive than it may appear. If two root-length assays differed by a constant factor, one of them would be redundant. Because they differ by different amounts on different compounds and rank compounds differently, the results are consistent with the two assays providing non-equivalent and potentially complementary information on herbicide toxicity, a possibility that remains to be tested rather than an established property of the assays. A battery containing both could therefore identify compounds for which the assays differ substantially in sensitivity, such as alachlor and, as a supporting observation, diuron, while also retaining compounds such as bentazone for which the *S. polyrhiza* assay was the more responsive. Whether this complementarity holds beyond the small number of compounds examined here requires a larger and chemically broader dataset, and the present dataset is not sufficient to recommend routine inclusion of both assays in a regulatory test battery.

### 4.5. Limitations

Five limitations bound the interpretation. First, a single endpoint was measured. No physiological, morphological or mechanistic data were collected, and statements about why the assays differ therefore lie outside what this dataset can support. Second, species and developmental starting condition are confounded by design; separating them would require the same species to be tested from both starting conditions, which is feasible for *S. polyrhiza*, where mature fronds and turion-derived plants can both be used [21], but was not done here. Third, organism loading differed between the assays, one frond against three turions per well, in accordance with the respective protocols; the two assays are therefore matched in exposure conditions but not in biological loading, and this difference is part of what is meant by an assay-level difference. Fourth, the primary comparison rests on only nine compounds, with one additional compound retained in the supporting set, so the absence of a significant rank correlation should be read as a failure to demonstrate concordance rather than as a demonstration of independence. Fifth, concentrations were nominal and not verified analytically; in small-volume test systems, pH drift and speciation can occur during exposure and may lead to underestimation of the toxicity of some substances [1]. For compounds whose highest nominal test concentrations approached or exceeded reported aqueous solubility, the nominal concentration cannot be assumed to represent the freely dissolved exposure concentration, and this uncertainty should be borne in mind when interpreting the magnitude of the between-assay differences for those compounds. The extent of this uncertainty differed among compounds. For most compounds, the definitive concentration ranges were below their reported aqueous solubilities, whereas the highest nominal *S. polyrhiza* concentrations exceeded reported solubility for diuron, butachlor and isoproturon, and the highest metolachlor concentration was close to its reported solubility. More importantly, the nominal EC_50_ estimates for butachlor and isoproturon (188.6 and 108.3 mg L^−1^, respectively) themselves exceeded their reported aqueous solubilities (approximately 20 and 65–70 mg L^−1^, respectively) and should therefore be regarded as nominal effect concentrations rather than analytically verified freely dissolved EC_50_ values. Compound-specific losses through photolysis, sorption, biodegradation or other processes during the 72 h static exposure cannot be quantified retrospectively. These exposure-related uncertainties limit mechanistic interpretation of the magnitude of individual assay differences, and the large nominal differences for butachlor and isoproturon in particular are interpreted cautiously. A separate DMSO solvent control was not included, although the maximum final DMSO concentration was below 0.1% (*v*/*v*), so a contribution of the solvent to the observed responses cannot be completely excluded.

## 5. Conclusions

Two duckweed toxicity tests that share the same measured endpoint, exposure duration, test format and initial root length of zero produced different herbicide EC_50_ patterns despite their close operational similarity. Across nine herbicides meeting the comparison-set precision criterion (*L. aequinoctialis* replicate EC_50_ CV < 40%), EC_50_ values differed by factors of 0.75 to 430, and significant concordance of potency ranking was not demonstrated; the composition of the primary comparison set was unchanged under a stricter 30% precision criterion. The tests differed in species, developmental starting condition, the biological origin of the measured root and organism loading, and the present data do not separate the contributions of these factors. Root re-growth in *L. aequinoctialis* and root development during turion germination in *S. polyrhiza* should therefore not be treated as interchangeable root-length tests, and comparable performance with a common reference toxicant should not be extended to other substance classes without direct evidence. The results are consistent with the two assays providing non-equivalent and potentially complementary information, but this possibility should be tested on a larger set of compounds and with the starting condition varied within a single species. For the two assay systems and the herbicides examined here, operational similarity did not imply toxicological equivalence.

## Figures and Tables

**Table 1:** Seventy-two-hour EC_50_ values (mg L^−1^, nominal) for inhibition of root length in *Spirodela polyrhiza* turions germinating in the test solution and in *Lemna aequinoctialis* root re-growth. For *S. polyrhiza*, values are single EC_50_ estimates; for *L. aequinoctialis*, values are means of three replicate EC_50_ estimates with 95% confidence intervals (Section 2.6). *R* is the ratio of the two EC_50_ values. Supporting-set rows are shaded. P, primary set; S, supporting set; –, not used quantitatively.

Herbicide	CAS No.	Target Site (HRAC)	*S. polyrhiza* EC_50_	*L. aequinoctialis* EC_50_ (95% CI)	CV (%)	*R*	Set
Alachlor	15972-60-8	VLCFA elongase (15)	96.160	0.401 (0.393–0.409)	0.83	239.8	P
Bentazone	25057-89-0	Photosystem II (6)	4.241	5.680 (3.880–7.480)	12.78	0.747	P
Butachlor	23184-66-9	VLCFA elongase (15)	188.6	2.560 (1.230–3.890)	20.87	73.69	P
Linuron	330-55-2	Photosystem II (5)	0.108	0.041 (0.031–0.051)	9.63	2.642	P
MCPA	94-74-6	Synthetic auxin (4)	4.636	2.100 (1.89–2.310)	3.96	2.208	P
Metolachlor	51218-45-2	VLCFA elongase (15)	50.010	18.150 (15.170–21.130)	6.60	2.755	P
Terbutryn	886-50-0	Photosystem II (5)	0.464	0.349 (0.330–0.368)	2.19	1.329	P
Diuron	330-54-1	Photosystem II (5)	4.395	0.019 (−0.002–0.040)	45.05	233.8	S
Penoxsulam	219714-96-2	Acetolactate synthase (2)	1.396	0.008 (0.003–0.012)	24.74	181.3	P
Atrazine	1912-24-9	Photosystem II (5)	>1	0.168 (0.139–0.197)	6.98	–	–
Simazine	122-34-9	Photosystem II (5)	>1	0.532 (0.036–1.028)	37.53	–	–
Isoproturon	34123-59-6	Photosystem II (5)	108.3	0.252 (0.213–0.291)	6.21	429.9	P
Thiobencarb	28249-77-6	VLCFA elongase (15)	>0.1	0.014 (0.004–0.025)	29.60	–	–

VLCFA, very-long-chain fatty acid; HRAC group numbers follow the 2020 global classification. For diuron, the negative lower 95% confidence bound results from the symmetric Student’s t-based interval and does not indicate a negative EC_50_.

## Data Availability

The data supporting the findings of this study are provided in the Supplementary Materials. Additional underlying data are available from the corresponding author on request.

## Supplementary Materials

The following supporting information can be downloaded at: https://www.mdpi.com/article/10.3390/toxics14090791/s1, Table S1: Definitive-test nominal concentration series and reported aqueous solubility of the 13 herbicides used in the two assays.; Table S2: Control root lengths obtained in the definitive herbicide experiments. Values are mean ± SD. For *S. polyrhiza*, values are based on four independent wells (*n* = 4), with the three turions within each well averaged before calculation. For *L. aequinoctialis*, values are based on three independent experimental sets (*n* = 3), with four root-length measurements averaged within each set.; Table S3: Potency ranks and rank concordance. Panel (a) gives the potency rank of each compound within each assay (rank 1 = lowest EC_50_, i.e. the most toxic compound in that assay), computed separately for the primary set of nine compounds and for the primary plus supporting set of ten. Panel (b) gives the corresponding rank-concordance statistics. Spearman rank correlation is the measure reported in the main text; Kendall rank correlation and the count of concordant and discordant pairwise orderings are given here because they use the same information in a different form and lead to the same conclusion. No correlation coefficient in this table is statistically significant at the 5% level. References [27,28,29,30,31,32,33,34,35] are cited in the Supplementary Materials.
