# Supplementary material for: Comparison of Herbicide Responses Between Root Re-Growth in Lemna aequinoctialis and Root Development During Turion Germination in Spirodela polyrhiza
Source: Toxics. 2026 Sep 7;14(9):791. doi: 10.3390/toxics14090791 (PMC13611772; doi:10.3390/toxics14090791)
Supplement: Supplementary file 1 [file toxics-14-00791-s001.zip › toxics-4518353-supplementary.pdf]

Supplementary Materials

Comparison of Herbicide Responses Between Root Re-Growth in *Lemna aequinoctialis* and Root Development During Turion Germination in *Spirodela polyrhiza*

Hojun Lee [1,2,3], Taejun Han [1,2,3] and Jihae Park [1,2,3,4,*]

[1] Marine@UGent Korea, 119-5, Songdomunhwa-ro, Incheon 21985, Republic of Korea; hojun.lee@ugent.be (H.L.); taejun.han@ghent.ac.kr (T.H.)
[2] Bio Environmental Science and Technology (BEST) Lab, Ghent University Global Campus, 119-5, Songdomunhwa-ro, Incheon 21985, Republic of Korea
[3] Department of Animal Sciences and Aquatic Ecology, Ghent University, Coupure Links 653-Block F, B-9000 Gent, Belgium
[4] Center for Green Chemistry and Environmental Biotechnology, Ghent University Global Campus, 119-5, Songdomunhwa-ro, Incheon 21985, Republic of Korea
* Correspondence: jihae.park@ghent.ac.kr

Table S1. Definitive-test nominal concentration series and reported aqueous solubility of the 13 herbicides used in the two assays.

**Herbicide**	*S. polyrhiza* **nominal (mg L^{-1})**	*L. aequinoctialis* **nominal (mg L^{-1})**	**Reported aqueous solubility (mg L^{-1})**	**Ref.**
Alachlor	6.25, 12.5, 25, 50, 100	0.625, 1.25, 2.5, 5, 10	240 (25°C)	[1]
Bentazone	6.25, 12.5, 25, 50, 100	6.25, 12.5, 25, 50, 100	500 (20°C)	[2]
MCPA	6.25, 12.5, 25, 50, 100	6.25, 12.5, 25, 50, 100	293,000 (pH 7)	[3]
Metolachlor	31.25, 62.5, 125, 250, 500	31.25, 62.5, 125, 250, 500	530 (20°C); 488 (25°C)	[4]
Butachlor	62.5, 125, 250, 500, 1000	3.125, 6.25, 12.5, 25, 50	20 (20°C)	[5]
Linuron	0.0625, 0.125, 0.25, 0.5, 1	0.0625, 0.125, 0.25, 0.5, 1	75 (25°C)	[6]
Terbutryn	0.625, 1.25, 2.5, 5, 10	0.625, 1.25, 2.5, 5, 10	25 (20°C)	[2]
Diuron	6.25, 12.5, 25, 50, 100	0.00625, 0.0125, 0.025, 0.05, 0.1	42 (25°C)	[7]
Penoxsulam	0.625, 1.25, 2.5, 5, 10	0.00625, 0.0125, 0.025, 0.05, 0.1	408 (pH 7, 20°C)	[8]
Atrazine	0.0625, 0.125, 0.25, 0.5, 1	0.0625, 0.125, 0.25, 0.5, 1	33 (25°C)	[2]
Simazine	0.0625, 0.125, 0.25, 0.5, 1	0.625, 1.25, 2.5, 5, 10	6.2 (pH 7, 20°C)	[8]
Isoproturon	62.5, 125, 250, 500, 1000	0.0625, 0.125, 0.25, 0.5, 1	65 (22°C); 70 (20°C)	[6,8]
Thiobencarb	0.00625, 0.0125, 0.025, 0.05, 0.1	0.00625, 0.0125, 0.025, 0.05, 0.1	30 (20°C)	[9]

Note: Each definitive test additionally included a 0 mg L^{-1} control. All test concentrations are nominal. The pH of the test medium was 6.9 ± 0.1. Reported aqueous solubility values are provided for comparison with the nominal concentration ranges and do not represent analytically verified exposure concentrations. Where solubility was strongly pH-dependent, values reported at or near pH 7 were used because they most closely correspond to the test-medium conditions. Aqueous solubility may vary with temperature, pH, medium composition, and other experimental conditions.

Table S2. Control root lengths obtained in the definitive herbicide experiments. Values are mean ± SD. For *S. polyrhiza*, values are based on four independent wells (n = 4), with the three turions within each well averaged before calculation. For *L. aequinoctialis*, values are based on three independent experimental sets (n = 3), with four root-length measurements averaged within each set.

Herbicide	*S. polyrhiza*, mm	*L. aequinoctialis*, mm
Alachlor	14.42 ± 1.30	27.34 ± 1.37
Bentazone	22.09 ± 2.09	23.77 ± 8.87
Butachlor	14.02 ± 1.67	35.41 ± 2.40
Linuron	18.61 ± 2.21	30.24 ± 3.87
MCPA	17.05 ± 1.55	12.68 ± 4.27
Metolachlor	16.49 ± 1.55	27.54 ± 2.15
Terbutryn	13.90 ± 4.61	26.09 ± 1.85
Diuron	14.72 ± 3.53	24.78 ± 5.28
Penoxsulam	11.56 ± 2.13	22.55 ± 1.35
Atrazine	9.44 ± 1.83	30.85 ± 2.19
Simazine	8.84 ± 1.06	30.02 ± 7.02
Isoproturon	13.60 ± 2.89	25.81 ± 1.75
Thiobencarb	13.17 ± 1.31	23.52 ± 4.87

Table S3. Potency ranks and rank concordance.

Panel (a) gives the potency rank of each compound within each assay (rank 1 = lowest EC_{50}, i.e. the most toxic compound in that assay), computed separately for the primary set of nine compounds and for the primary plus supporting set of ten. Panel (b) gives the corresponding rank-concordance statistics. Spearman rank correlation is the measure reported in the main text; Kendall rank correlation and the count of concordant and discordant pairwise orderings are given here because they use the same information in a different form and lead to the same conclusion. No correlation coefficient in this table is statistically significant at the 5% level.

(a) Potency ranks

Herbicide	Rank, *S. polyrhiza* (n = 9)	Rank, *L. aequinoctialis* (n = 9)	|Δ| (n = 9)	Rank, *S. polyrhiza* (n = 10)	Rank, *L. aequinoctialis* (n = 10)
Linuron	1	2	1	1	3
Terbutryn	2	4	2	2	5
Bentazone	4	8	4	4	9
MCPA	5	6	1	6	7
Metolachlor	6	9	3	7	10
Alachlor	7	5	2	8	6
Butachlor	9	7	2	10	8
Isoproturon	8	3	5	9	4
Penoxsulam	3	1	2	3	1
Diuron	–	–	–	5	2

Diuron belongs to the supporting set and is ranked only in the ten-compound comparison.

(b) Rank-concordance statistics

Comparison set	*n*	Spearman ρ (p)	Kendall τ (p)	Pairs	Concordant / discordant
Primary set	9	0.433 (0.244)	0.278 (0.358)	36	23 / 13
Primary + supporting set	10	0.430 (0.214)	0.289 (0.291)	45	29 / 16
Primary set, 30% CV limit	9	0.433 (0.244)	0.278 (0.358)	36	23 / 13

A pair of compounds is concordant when the two assays place them in the same order of potency and discordant when the order is reversed. Under a stricter 30% CV limit the primary comparison set is unchanged, because all nine primary-set *L. aequinoctialis* EC_{50} estimates have coefficients of variation below 30%; the corresponding row is therefore identical to the primary set. Statistics were computed in Python 3.12 with SciPy.

References

1. Chesters, G.; Simsiman, G.V.; Levy, J.; Alhajjar, B.J.; Fathulla, R.N.; Harkin, J.M. Environmental fate of alachlor and metolachlor. *Rev. Environ. Contam. Toxicol.* **1989**, *110*, 1–74.
2. Yalkowsky, S.H.; He, Y.; Jain, P. *Handbook of Aqueous Solubility Data*, 2nd ed.; CRC Press: Boca Raton, FL, USA, 2010.
3. Health Canada. *Guidelines for Canadian Drinking Water Quality: Guideline Technical Document, 4-Chloro-2-methylphenoxyacetic Acid (MCPA)*; Health Canada: Ottawa, ON, Canada, 2022.

4. Wauchope, R.D.; Buttler, T.M.; Hornsby, A.G.; Augustijn-Beckers, P.W.M.; Burt, J.P. The SCS/ARS/CES pesticide properties database for environmental decision-making. *Rev. Environ. Contam. Toxicol.* **1992**, *123*, 1–155.
5. Tomlin, C. (Ed.) *The Pesticide Manual: A World Compendium*, 11th ed.; British Crop Protection Council: Farnham, UK, 1997.
6. O'Neil, M.J. (Ed.) *The Merck Index: An Encyclopedia of Chemicals, Drugs, and Biologicals*, 15th ed.; Royal Society of Chemistry: Cambridge, UK, 2013.
7. U.S. Department of Agriculture, Agricultural Research Service. *ARS Pesticide Properties Database*; USDA-ARS: Beltsville, MD, USA, 1995.
8. MacBean, C. (Ed.) *The e-Pesticide Manual*, 15th ed.; Version 5.1; British Crop Protection Council: Alton, UK, 2008–2010.
9. Australian and New Zealand Environment and Conservation Council; Agriculture and Resource Management Council of Australia and New Zealand. *Australian and New Zealand Guidelines for Fresh and Marine Water Quality*; ANZECC & ARMCANZ: Canberra, Australia, 2000.
